# The spatiotemporal differentiation and influencing factors of urban vitality in the border areas of China

**DOI:** 10.1371/journal.pone.0330856

**Published:** 2025-09-03

**Authors:** Fengwei Ai, Qingshan Yang

**Affiliations:** 1 School of Geographical Sciences, Northeast Normal University, Changchun, Jilin Province, China; 2 School of Tourism Geography and Historical Culture, Hulunbuir University, Hulunbuir, Inner Mongolia Autonomous Region, China; Universiti Kebangsaan Malaysia, MALAYSIA

## Abstract

Based on the concept of urban vitality and the unique geographical location of border areas, an evaluation framework for urban vitality in border areas was constructed from four dimensions: economy, society, openness, and culture. The entropy weight method was used to calculate the urban vitality levels of 45 border cities in 2000, 2010, 2019, and 2022, and the influencing factors were quantitatively analyzed. The results indicated that from 2000–2022, the vitality level of China’s border cities continued to rise, from 1.38 to 6.57 with an average annual growth rate of 7.35%. The southwestern border region exhibited the highest growth rate, which was 5.3 times greater than that of the western border region with the slowest growth. Spatially, the areas with high urban vitality shifted from the northeast area to the southwest area, with key vitality centers emerging in Mudanjiang (northeast), Baotou (north), Bortala Mongolian Autonomous Prefecture (northwest), and Chongzuo (southwest). Among the influencing factors, openness vitality was found as the primary internal constraint on urban vitality, while border policies and innovation capabilities were identified as the most significant external drivers. This research aims to provide both theoretical insights and empirical evidence to guide the enhancement of border city vitality, contributing to the broader global discourse on border region development.

## 1. Introduction

With the acceleration of globalization and the continuous deepening of regional economic integration, border cities, as gateways for cross-border trade and investment, hold a significant strategic position in international cooperation and economic development [1]. China, with a land border extending over 22,000 kilometers and adjoining 14 countries including Russia, Mongolia, North Korea, Vietnam, Laos, Myanmar, India, Pakistan, and others, bestows China’s border areas with unique geographical and strategic advantages, making them the forefront of the country’s opening-up to the outside world [2]. Driven by the in-depth implementation of the “Belt and Road” Initiative and the 14th Five-Year Plan (2021–2025), the high-quality development of border cities has received unprecedented attention [3]. Urban vitality in these regions plays a critical role not only in attracting talent and capital but also in enhancing China’s overall competitiveness on the global stage [4]. However, these areas continue to face multiple challenges, such as the weak economic foundation, uneven degree of opening up to the outside world and insufficient innovation capability, which have significantly limited the vitality enhancement of border cities [5–7]. It raises a series of issues: How does the urban vitality of border cities manifest? What are the key factors shaping urban vitality in these areas? How can we leverage unique location advantages to promote high-quality development of cities in border areas? All of these require urgent study.

Over the past decades, scholars have conducted extensive discussions on urban vitality, reaching several important findings. First, urban vitality is widely acknowledged as a multidimensional concept encompassing economic activity, social interaction, spatial structure, and cultural dynamics [8,9]. Second, existing studies have examined factors such as urban morphology, land-use diversity, public space accessibility, and policy interventions, which significantly influence urban vitality levels [10,11]. Third, a variety of measurement methods have been proposed and refined, including the obstacle level model, fuzzy comprehensive evaluation, semantic analysis, and data-driven approach, providing valuable tools for optimizing urban vitality [12–15]. However, the vast majority of research has focused on urban internal spaces, such as urban streets, central urban areas, and public spaces [16,17], while the research areas mostly focused on developed cities and core economic zones [18–23]. In contrast, border cities remain understudied, both theoretically and empirically, despite their increasing geopolitical and developmental importance.

To address the research gap, this research constructed an evaluation framework for urban vitality applicable to border areas from four dimensions: economy, society, openness to the outside world, and culture. Then, the entropy weight method was applied to quantitatively measure the urban vitality of China’s border areas in 2000, 2010, 2019, and 2022. By applying this method to a large-scale case study of 45 cities, the study provided a robust and generalizable model for evaluating urban vitality, which can be applied to similar border cities worldwide. To further examine the factors affecting vitality development, the obstacle degree model was employed to identify internal barriers, while multiple linear regression analysis was used to assess the influence of external factors. By this way, this research could offer targeted policy recommendations to local governments and urban planners, emphasizing the need to focus on innovation capacity building, improving external connections, and fostering cultural exchanges to boost urban vitality in border regions. These recommendations are not only applicable to China but can also be adapted to other countries with border cities facing similar challenges.

## 2. Urban vitality of border cities

### 2.1 Definition of urban vitality

Urban vitality has long been an essential concept in urban studies. Jacobs [24] first proposed the concept of urban vitality in “The Death and Life of American Big Cities” arguing that urban vitality originates from the interaction between human activities and urban space, which is closely related to urban diversity and emphasized the interactive relationship between urban vitality and urban space; Lynch [9] further developed this notion, defining urban vitality as the ability of urban systems to maintain their internal survival, growth, and development, which was a dimension of urban form that supports human life and social functions. Both Jacobs and Lynch focused on urban space, but Lynch more focused on the impact of urban form on residents’ perception and behavior, while Jacobs stressed the importance of diversity in urban blocks. Bentley [10] described urban vitality as the degree to which it influences specific places and accommodates different functions, emphasizing the importance of functional diversity. Some Chinese scholars also gave their understandings based on Chinese context. Jiang conceptualized urban vitality as a city’s capacity to offer humane living conditions, categorizing it into economic, social and cultural vitality [25]; Tong [3] argued that urban texture is the key to stimulating urban vitality, promoting a reciprocal relationship between material environment and social functions; Wang [12] expanded this view by highlighting that urban vitality not only includes the explicit vitality of physical space, but also the implicit vitality of digital space. Another concept closely related to urban vitality is urban conviviality, which refers to the harmonious coexistence, positive interaction, and mutual respect among diverse social groups within urban environment [26]. Urban conviviality is an important component of urban vitality, especially in border cities, where multiculturalism is often a defining feature, and the ability to integrate diverse populations can greatly enhance urban vitality.

In the context of globalization, border regions have evolved from peripheral zones into strategic nodes within global urban networks [27,28]. Borders today function less as rigid lines of division and more as dynamic spaces of interaction, innovation, and competition. In regions with enhanced cross-border mobility—such as parts of Europe and Southeast Asia—cooperative frameworks have reshaped spatial development patterns [29]. However, this pattern is not uniform. Some countries, extensive land borders coexist with low urban vitality due to factors like inadequate infrastructure, political instability, or economic isolation [30,31]. Therefore, mere geographic proximity to a border is insufficient to generate urban vitality. The development of vibrant border cities relies on the synergistic integration of internal capacities (such as economic strength, innovation capacity) and external linkages (such as cross-border trade, policy support).

China’s border cities offer a distinctive case. Over the past decades, with initiatives such as the “Belt and Road” Initiative and regional cooperation platforms (such as the China-ASEAN Free Trade Area), China’s border regions have been deeply integrated into global and regional economic systems [32,33]. Unlike many counterparts, China’s borders are characterized by strong state-led infrastructure investments and relatively stable geopolitical relations with most neighboring countries. These characteristics offer a unique lens through which to examine the interaction between border proximity and urban vitality, particularly within the broader context of globalization and regional integration. Therefore, research on China’s border cities carries not only regional significance but also contributes to a deeper theoretical understanding of how such cities can be repositioned as engines of development in the evolving global landscape.

Based on the above understanding, this research defines the vitality of border cities as the ability of effectively integrate and utilize resources, to promote the vigorous development of economy, society, openness, culture and other aspects in the special geographical location and multicultural background of border cities. Through continuous innovation and adaptive adjustment in practice, urban vitality contributes to promote cooperation, exchange with neighboring countries or regions, and enhance regional comprehensive competitiveness and sustainable development capabilities.

### 2.2 Evaluation framework of urban vitality

#### 2.2.1 Dimensions of urban vitality.

Based on the definition proposed, a total of 16 secondary indicators are selected to construct an evaluation framework for urban vitality from four dimensions: economy, society, openness and culture. This framework provides a scientifically grounded and comprehensive approach to assessing the vitality of border cities:

(1) Economic vitality. Economic vitality refers to the mechanism and capacity of a country or region to sustain economic growth [34]. Jin asserted that economic vitality is the ability and potential in the process of urban vitality development [14]. Therefore, this research considers the economic vitality as an essential aspect of urban vitality.
(2) Social vitality. Social vitality reflects the level of interaction, mobility and participation of residents within urban spaces, serving as a key indicator of social development, public service capacity, and quality of life [17]. Education, healthcare, welfare provision, and employment conditions collectively underpin the stability and dynamism of the social system [35].
(3) Openness vitality.This reflects the level of development of outward-oriented economic activities in border cities [36]. Openness vitality is a factor of urban development formed by relying on the unique geographical location of border areas. Promoting the outward oriented economy of border cities, actively participating in international exchanges and competition, and stimulating the vitality of border cities in opening up to the outside world, can drive the development of regional economy in border cities.
(4) Cultural vitality is a strong spiritual and dynamic support for urban development [37]. It reflects the degree of participation of cities in the processes of cultural storage, exchange, dissemination and creation. Enhancing cultural vitality not only improves the attractiveness of a city, but also showcases its personality and creativity.

#### 2.2.2 Indicator selection.

Economic vitality. The proportion of the secondary and tertiary industries to GDP reflect a region’s transformation towards a more efficient and competitive economic structure during the process of economic modernization. By observing changes in industrial structure, the trend of economic vitality can be more accurately captured. Moreover, local general public budget revenue indicates the speed and quality of economic growth and reveal the structure of regional economic development as well as the effectiveness of economic policies. GDP per Capita is also a common indicator reflecting the local economic development, and the total profit of large-scale enterprises can reflect regional adaptability and innovation capability in a fiercely competitive market environment.

Social vitality. Social fixed assets investment not only reflects the improvement of social infrastructure, public service development and social welfare, but reveals the stability, harmony and sustainability of society. The number of hospital beds reflects the allocation level of medical resources and the accessibility of medical services, which is indicative of society’s investment in and fairness in providing public services, especially for remote areas. The total retail sales of consumer goods reflect the consumption level of residents in a region. An increase in retail sales is often closely linked to the modernization process, technological innovation, and changes in consumption patterns, reflecting society’s innovation capacity and flexibility in adapting to market changes. The registered urban unemployment rate at the end of the year reflects the economic health of society, which indirectly affects the stability and vitality of society.

Openess vitality. The import and export volume directly shows the strength of the connection between the border city and the international market. It can comprehensively reflect the city’s position in international trade, as well as its level of activity in the global supply chain and market. International tourism receipts reflect the international attractiveness of border cities, the frequency of cross-border exchanges, and the level of outward-oriented development of the city’s economy, revealing the city’s economic extroversion, cultural soft power and cross-border cooperation. The actually utilized foreign capital reflects the level of openness, market attractiveness, and international economic integration of border cities, as well as their ability to attract international capital, promote industrial development, and drive sustainable socio-economic growth.

Cultural vitality. The per capita collection of public library books indicates the acquisition of knowledge, information and culture by members of society, especially for border cities. Due to the unique geographical location, border areas are often the places where diverse cultures intersect. As an important platform for cultural exchange, public libraries promote interaction and integration among residents from different cultural backgrounds, enhancing cultural inclusiveness and innovation. Technological progress provides new tools, innovative dissemination platforms, and more efficient production methods for the cultural industry. Border cities not only enhance the competitiveness of the cultural industry through investment in technology, but also foster cross-regional and cross-cultural communication, increasing the influence of local culture in the context of globalization. Border cities, being at the forefront of international exchanges, benefit from increasing education expenditure, which enhances their ability for cultural exchange and interaction, promoting the internationalization and modernization of local culture. The number of college students in higher education institutions is also a crucial source of cultural innovation, social vitality and ideological vitality. By attracting a large number of college students, border cities can enhance their cultural innovation capabilities, the appeal of educational resources, and the diversity of cultural activities, thus revealing the potential and vibrancy of their cultural development. The specific indicator system is shown in Table 1.

**Table 1:** Evaluation index system of urban vitality in border areas of China.

System	Indicator layer	Explanation of indicators	Unit	Weight
**Economic vitality**	The proportion of the secondary and tertiary industries to GDP A1	The rationality of characterizing industrial structure	%	0.0375
	Local general public budget revenue A2	The direct reflection of the level and vitality of local economic development	100 million yuan	0.0700
	GDP per Capita A3	Reflecting on the quality of life of the people	Yuan per capita	0.0581
	Total profit of large-scale enterprises A4	Measuring the level of industrialization	100 million yuan	0.0690
**Social vitality**	Social fixed assets investment B1	Measuring the investment effectiveness of local governments	100 million yuan	0.0667
	Number of hospital beds B2	The medical supply capacity and medical security status of the city	Beds	0.0288
	Total retail sales of consumer goods B3	Characterize the consumption level of residents	100 million yuan	0.0566
	Registered urban unemployment at the end of the year B4	Indicators for measuring employment	ten thousand people	0.0327
**Openess vitality**	Import and export volume C1	Reflecting the overall scale and development level of foreign trade	USD 100 million	0.0940
	Total import and export volume/GDP C2	Indicator for measuring foreign trade relative to the overall economic scale	%	0.0622
	International Tourism Receipts C3	Indicators for measuring the level of international inbound tourism development	USD 100 million	0.1021
	Actually utilized foreign capital C4	Indicating the driving role of foreign capital in economic development	USD 100 million	0.1047
**Cultural vitality**	Per capita public library collection D1	The completeness of public cultural facilities	Volume/person	0.0466
	Technology expenditure D2	The level of investment in technology by cities	100 million yuan	0.0594
	education expenditure D3	The level of investment in education by cities	100 million yuan	0.0443
	Number of college students enrolled in higher education institutions D4	Indicate the degree of concentration of high-quality talents	ten thousand people	0.0673

## 3. Methodology

### 3.1 Study area

**Fig 1:**
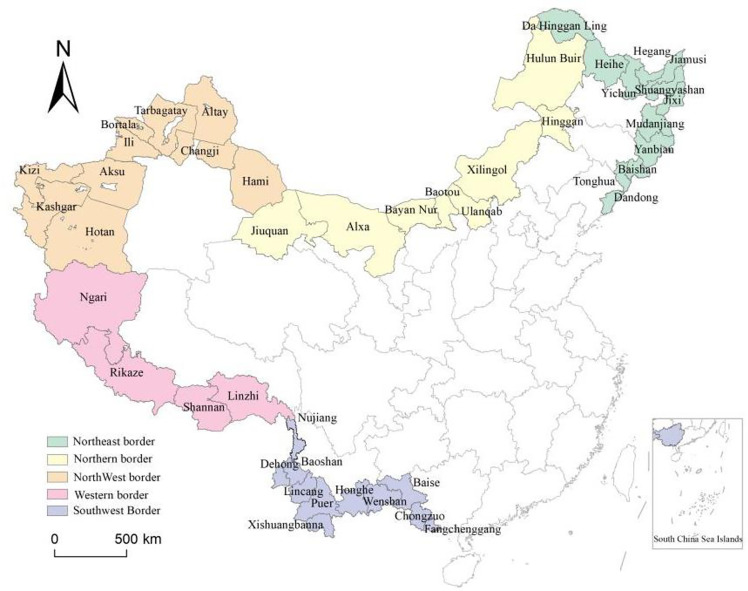
The border cities in China.

China’s land border areas encompass nine provinces and autonomous regions, including Liaoning, Jilin, Heilongjiang, Inner Mongolia, Gansu, Xinjiang, Tibet, Yunnan and Guangxi, and a total of 45 prefecture-level municipal administrative regions. To facilitate the further analysis, this study divides China’s border areas into five regions [38], namely, the northeast border (comprising the border areas of the three northeastern provinces; refer to the green area in Fig 1), the northern border (comprising the border areas of Inner Mongolia Autonomous Region and Gansu Province; refer to the yellow area in Fig 1), the northwest border (comprising the border areas of Xinjiang Uygur Autonomous Region; refer to the orange area in Fig 1), the western border (comprising the border areas of Tibet Autonomous Region; refer to Fig 1 for the pink area), and the southwest border (comprising the border areas of Guangxi Zhuang Autonomous Region and Yunnan Province; refer to Fig 1 for the purple area).

The northeastern border area borders Russia and North Korea, with a relatively long border. The terrain is dominated by mountains, hills and plains, including the Little Xing’an Mountains, the Changbai Mountains, and the Songliao Plain, with temperate monsoon climate and cold temperate climate. In 2022, the GDP of this region was 0.75 trillion yuan, accounting for 0.6% of the national total, with a total population of 16 million and a GDP per Capita of 47,000 yuan, which is lower than the national average (the national GDP per Capita is 87,400 yuan). The total import and export volume was 0.09 trillion yuan, accounting for 3% of the national total, and its foreign trade volume was lower than that of coastal provinces.The northern border region spans across northern China and borders Mongolia and Russia. The terrain is dominated by plateau, grasslands, deserts and hills, with an arid climate and temperate continental conditions. In 2022, the GDP of this region was 1.05 trillion yuan, accounting for 0.8% of the national total, with a total population of 12 million people and a GDP per Capita of 90,000 yuan. It is the only border area where GDP per Capita is higher than the national average, with a total import and export volume of 0.1 trillion yuan, accounting for 3% of the national total import and export volume.

The northwest border region borders multiple countries, including Kazakhstan, Tajikistan, Kyrgyzstan, Uzbekistan, Russia, Mongolia, Afghanistan, Pakistan and India, with the longest border line. It features typical basin mountain terrain, such as Tarim Basin, the Junggar Basin, and Tianshan Mountains with scarce precipitation and many deserts. In 2022, the GDP of the region was 1.14 trillion yuan, accounting for 0.9% of the national total. The total population was 18 million people, and the GDP per Capita was 74,000 yuan, slightly below the national average. The total import and export volume was 0.14 trillion yuan, accounting for 4.9% of the national total import and export volume.

The western border region borders countries such as India, Bhutan, Nepal, and Myanmar. The terrain is complex, dominated by the Qinghai-Tibet Plateau and the Himalayas, with the highest elevation on the roof of the world. The climate is cold, mainly plateau climate, with thin air and low precipitation. In 2022, the GDP of the region was 0.09 trillion yuan, accounting for 0.07% of the national total, with a total population of 2 million people and a GDP per Capita of 67,000 yuan, which is lower than the national average. The total import and export volume was 0.003 trillion yuan, accounting for 0.1% of the national total import and export volume, making it the border area with the lowest foreign trade.

The southwestern border region borders Myanmar, Laos, and Vietnam, characterized by active border trade. The terrain mainly consists of mountains, plateaus and river valleys, with the Lancang-Mekong River basin running through the region. The climate is warm, mainly consisting of subtropical monsoon climate and tropical climate. It is the core region of China ASEAN Free Trade Area. In 2022, the GDP of the region was 1.29 trillion yuan, accounting for 1% of the national total, with a total population of 25 million people and a GDP per Capita of 54,000 yuan, which is lower than the national average. The total import and export volume was 0.47 trillion yuan, accounting for 16.5% of the national total, making it the border area with the highest foreign trade volume.

### 3.2 Research methods

To explore the urban vitality of China’s border areas, this research first applies the entropy method to comprehensively measure the vitality from four dimensions: economic vitality, social vitality, openness vitality and cultural vitality. Then to investigate the factors hindering the development of urban vitality in border region, this study adopts an obstacle degree model to identify the internal obstacle factors restricting the development of urban vitality. Finally, multiple linear regression analysis is employed to examine the mechanisms through which external influencing factors, such as neighboring countries’ development strength, market opportunities, border policies, innovation capabilities, ecological environment, and city size, affect urban vitality, thereby complementing the exploration conducted through the obstacle degree model. Through the above efforts, the study seeks to achieve a comprehensive evaluation of the vitality of cities in China’s border areas, reveal the key factors that affect these urban vitality, and provide scientific basis for policy-making.

#### 3.2.1 Entropy weight method.

This research used fact data to avoid the subjective bias of evaluators and an objective assignment method to determine weights, thereby obtaining more accurate and reasonable evaluation results [39]. The entropy weight method helps to avoid randomness, overcome the problem of information overlap between multiple variables, and objectively and accurately evaluate the research object.

Given the complexity and diversity of evaluation indicators, it is necessary to first standardize a total of 2,880 data from 16 indicators before conducting statistical methods. The standardization process is as follows:


$$\text{Positive indicators}:y_{ij}=\frac{x_{ij}-x_{jmin}}{x_{jmax}-x_{jmin}\text{\textbackslash{} }}+\text{0}.\text{0001};\text{ Reverse indicators}:\;y_{ij}=\frac{x_{max}-x_{ij}}{x_{jmax}-x_{jmin}\;}+\text{0}.\text{0001\textbackslash{} \textbackslash{} }$$ (1)

Where *y*_*ij*_ is the standardized value of the index and *x*_*ij*_ is the initial value of the index.

Step 2 is to determine the indicator weights:


$$\begin{array}{c} P_{ij}=\frac{y_{ij}}{\sum_{i=\text{1}}^{n}y_{ij}};\;e_{j}=-\frac{\text{1}}{\text{ln}\;n}\sum_{i=\text{1}}^{n}P_{ij}\;\text{ln}\;P_{ij};\;d_{j}=\text{1}-e_{j};W_{j}=\frac{d_{j}}{\sum_{i=1}^{n}d_{j}} \end{array}$$ (2)

Among them, *P*_*ij*_ represents the proportion of indicators; *e*_*j*_ is the information entropy value; *d*_*j*_ is the coefficient of indicator difference; *W*_*j*_ is the weight of the jth indicator; *n* is the number of cities.

Then based on the weights derived, the urban vitality index of border cities could be obtained:


$$S={\sum {\mathrm{\backslash nolimits}}_{j=\text{1}}^{n}W_{j}y}_{ij}$$ (3)

#### 3.2.2 Obstacle degree model.

This research aims to analyze the impact of internal obstacles on the local urban vitality development by employing obstacle degree model. This model not only provides a deeper understanding of the spatiotemporal evolution of urban vitality in border areas, but also offers valuable insights for decision-making [40]. The specific process involves “indicator deviation degree” and “obstacle degree” for analysis and diagnosis [41]. The formula is as follows:


$$I_{ij}=1-x_{ij};\;C_{ij}=\frac{W_{ij}\times I_{ij}}{\sum_{j=1}^{n}w_{ij}\times I_{ij}}\;\times 100\%$$ (4)

*I*_*ij*_ represents the deviation degree of the indicator, which measures the difference between the actual value and the ideal value of the *j*th indicator in the *i*th criterion layer; the indicator weight calculated by formula (2) for *W*; *C*_*ij*_ represents the degree of impact of the *j*th indicator in the *i*th criterion layer on urban vitality, and the larger the *C* value, the greater the impact of this factor on urban vitality in China’s border areas, and the stronger the obstacle degree.

#### 3.2.3 Multiple linear regression analysis.

This research builds on the existing research on urban vitality and ultimately selects neighboring countries’ development strength [42], market opportunities [42], border policies [38], innovation capabilities [43], ecological environment [44] and city size [21] to explore the impacts of external factors on urban vitality development through multiple linear analysis (see Table 2). Multiple linear regression analysis is a statistical method used to assess the importance of the independent variable’s influence on the dependent variable and predict the future trend of the dependent variable [45]. Through multiple linear regression, the relationship between external influencing factors and the level of urban vitality can be analyzed, and the dominant external factors that affect changes in urban vitality can be identified.

**Table 2:** Variable description.

Influence factor	Proxy variable	Data sources
I**nnovation capabilities**	Number of patent authorizations	China National Intellectual Property Administration
E**cological environment**	Per capita water resources	Statistical yearbook
B**order policies**	Assignment	1The state council approves class I port for opening up to the outside world 2 Key development and opening experimental zone 3 Border economic cooperation zone 4 Cross-border economic cooperation zone(To measure the extent to which a city receives national policy support, assign a value of 1–4 based on the above 4 policy conditions, with each item corresponding to 1 point)
N**eighboring countries’ development strength**	Composite index	World Bank
M**arket opportunities**	Per capita of neighboring countries/GDP per Capita of regions	World Bank
C**ity size**	Population density	Statistical yearbook

The mathematical model is expressed as:


$$\;Y=\mu +\beta_{1}x_{1}+\beta_{2}x_{2}+...+\beta_{n}x_{2n}+\varepsilon$$ (5)

where Y is the dependent variable (urban vitality); μ is a constant term; *β*_*n*_ represents the regression coefficients of each variable; *x*_*n*_ represents the independent variable, and *ε* is the error term.

After determining the multiple linear regression model, a t-test was conducted to test the significance of each regression coefficient and analyze whether the impact of each independent variable on the dependent variable is statistically significant.

### 3.3 Data sources

This study selects the years 2000, 2010, 2019, and 2022 for statistical analysis, considering three key factors. First, the introduction of the “prospering frontier and enriching people” policy in 1999 marked a significant shift in border region development. Second, the implementation of supportive policies for these regions in 2009 further enhanced their development. Finally, the impact of the COVID-19 pandemic during 2019 and 2022 introduced new challenges and changes that need to be accounted for in the analysis.

The data primarily comes from the Statistical Yearbooks of Chinese Cities in 2001, 2011, 2020, and 2023, as well as the statistical yearbooks of nine provinces and autonomous regions along the border. A small amount of data is supplemented by the national economic and social development statistical bulletins of relevant prefecture-level cities and World Bank.

In this study, some data was missing, mainly occurring in certain years when statistical analysis was unavailable. To ensure the rationality and accuracy of data supplementation, the linear interpolation method was employed. During the interpolation process, for each missing data point, the linear calculation was performed using adjacent data points above and below to estimate the missing value. Interpolation ensures a smooth transition of data and avoids analysis bias caused by missing data.

## 4. Result

### 4.1 The spatiotemporal differentiation of urban vitality in China’s border areas

#### 4.1.1 The spatiotemporal evolution characteristics of urban vitality in China’s border areas.

Based on the indicator system (as shown in Table 1), the urban vitality of 45 border prefecture-level cities in China was calculated and divided into five levels: high, higher, medium, lower, and low vitality using the natural breaks method (Table 3).

**Table 3:** Urban vitality level in border areas of China in 2000–2022.

Vitality level	Range				Number of cities				The proportion of cities/cumulative proportion%			
	2000	2010	2019	2022	2000	2010	2019	2022	2000	2010	2019	2022
**Low**	0.01-0.02	0.02 −0.03	0.04-0.08	0.04-0.08	10	5	11	8	22/22	11/11	25/25	18/18
**Lower**	0.02-0.03	0.03 −0.07	0.08-0.13	0.08-0.12	15	18	11	11	34/56	40/51	25/50	25/43
**Medium**	0.03-0.04	0.07 −0.11	0.13-0.20	0.12-0.15	13	12	12	10	29/85	27/78	27/77	22/65
**Higher**	0.04-0.06	0.11 −0.19	0.20-0.27	0.15-0.20	6	7	8	9	13/98	16/94	17/94	20/85
**high**	0.06-0.10	0.19 −0.42	0.27-0.43	0.20-0.40	1	3	3	7	2/100	6/100	6/100	15/100

**Fig 2:**
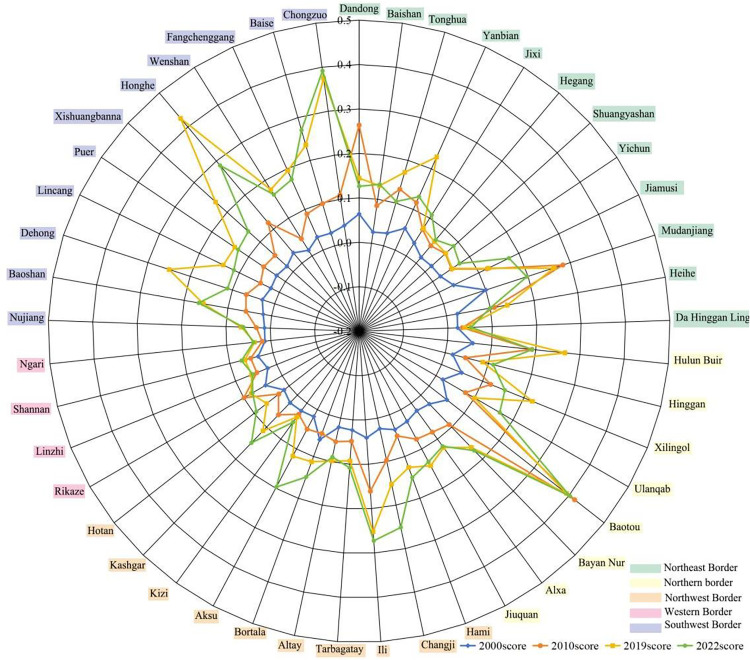
Urban Vitality Index in border areas of China in 2000–2022.

From the perspective of temporal evolution (as shown in Fig 2), the vitality of China’s border areas has continuously risen from 1.38 in 2000 to 6.57 in 2022, the average annual growth rate of urban vitality index was 7.35%, with the period from 2010 to 2019 marking a peak. From 2000 to 2022, the average urban vitality index increased as follows: the Northeast border area rose from 0.04 to 0.11, the Northern border area increased from 0.03 to 0.18, the Northwest border area increased from 0.03 to 0.15, the Western border area grew from 0.03 to 0.06, and the Southwest border area rose from 0.02 to 0.18. The fastest-growing region was the southwestern border area, with urban vitality increasing 5.3 times compared to the lowest Western border area.

**Fig 3:**
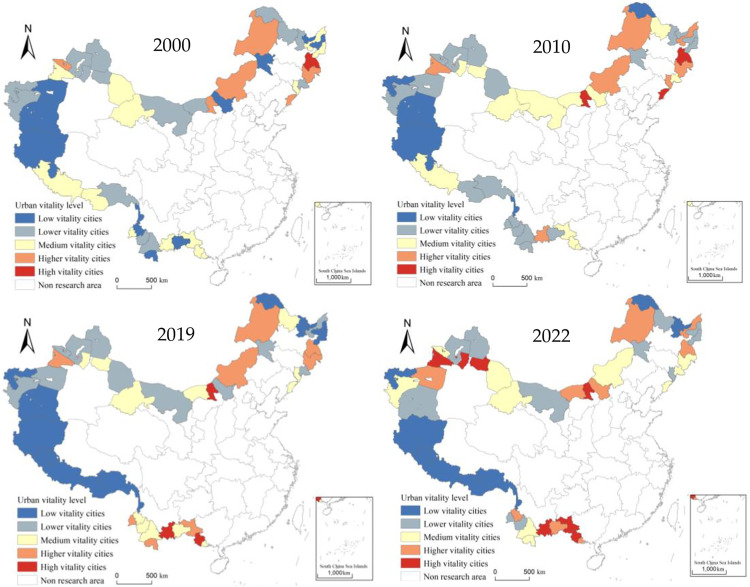
Spatial-temporal pattern of urban vitality in border areas of China in 2000–2022.

From a spatial distribution perspective (See Fig 3), there are significant differences in urban vitality among border areas. The area with the highest urban vitality index in 2000 was Mudanjiang City in the northeast border region, which shifted to Baotou City in the northern border region by 2022. Due to geographical and infrastructure limitations, the least vibrant region has consistently been the Ali area in the western border. In terms of the change in urban vitality, the region with the fastest growth was the southwestern border area.The urban vitality of Chongzuo City, Honghe Hani and Yi Autonomous Prefecture, and Dehong Dai and Jingpo Autonomous Prefecture grew rapidly, and a new border core city needs to be formed, which was benefited from the increasingly close trade ties between this region and ASEAN countries, policy support, and infrastructure construction. The region with the largest decline in urban vitality was the northeastern border area, which was primarily affected by multiple factors such as difficulties in economic structural transformation, population loss, and insufficient policy support. Overall, the urban vitality of the border area was gradually shifting from the northeast border area to the southwest border area.

Overall, cities with high urban vitality were mainly concentrated in resource-based cities with well-developed port facilities and strong economic foundations. The northeastern border region was led by Mudanjiang City, which is located in the central area of the Northeast Asian Economic Circle and hosting four first-class national ports. It was also the only “demonstration city of friendly cooperation between China and Russia ” in China. The northern border region was led by Baotou City, which is the largest industrial city in Inner Mongolia, rich in natural resources such as coal and rare earths. Relying on resources, Baotou formed a relatively complete industrial system with high technological innovation capabilities, providing strong support for economic development. The northwestern border region was led by Bortala Mongolian Autonomous Prefecture, which was an important key node of the “Silk Road Economic Belt” both domestically and internationally. This region was also connected by the second Eurasian bridge, with Alashankou ranking among the top in terms of cargo volume and import/export trade in the country. In the southwestern border region, the spatial differences in urban vitality were not significant, with Chongzuo City as the leader. At the same time, the urban vitality level of Dehong Dai Jingpo Autonomous Prefecture and Honghe Hani and Yi Autonomous Prefecture was relatively high. Chongzuo City was the border city with the most ports in China and also had the most convenient land transportation from China to ASEAN, with outstanding geographical advantages.

#### 4.1.2 The spatiotemporal evolution characteristics of urban vitality subsystems in China’s border areas.

**Fig 4:**
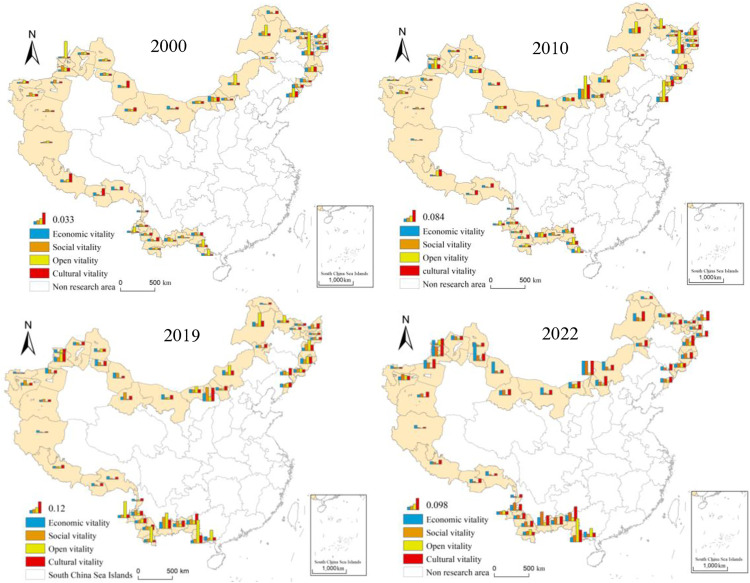
The level of urban subsystems in border areas of China in 2000–2022.

**Fig 5:**
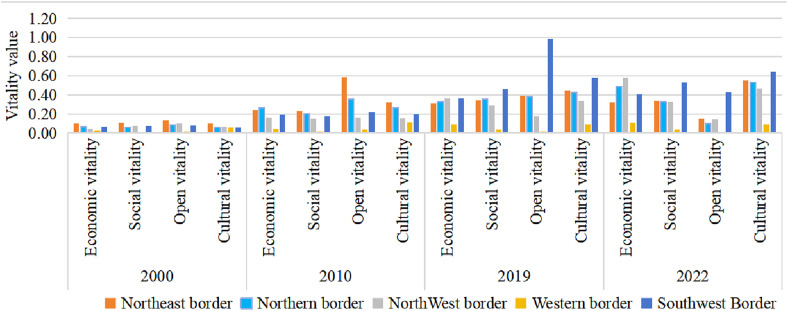
The Changes of subsystem vitality levels in border areas of China in 2000–2022.

Figs 4 and 5 clearly illustrate the overall upward trajectory of the four subsystems of urban vitality from 2000 to 2022, despite persistent regional disparities. Economic vitality grew most rapidly in the northern, northwestern, and southwestern border regions, led respectively by Baotou, Bortala Mongol Autonomous Prefecture, and Fangchenggang. Social vitality developed more slowly in the western border region due to the limitations in transportation, education, and healthcare infrastructure. The center of openness vitality gradually shifted from the northeast to the southwest. In 2000, the northeastern border region had the highest level of openness vitality, with cities such as Mudanjiang and Dandong standing out due to their high-grade ports. Since 2010, driven by the implementation of the “prospering frontier and enriching people” initiative and the “Belt and Road” Initiative, the level of openness in the southwest has improved rapidly, with Fangchenggang and Chongzuo emerging as new frontiers of international cooperation. Cultural vitality increased at a more moderate pace but showed clear spatial clustering, with the southwestern region experiencing notable growth by capitalizing on its rich ethnic minority cultural resources.

Overall, economic and openness vitality exhibited greater fluctuations due to the influence of policies and geographic advantages, whereas social and cultural vitality improved more steadily, relying on long-term investment and public service development. In the future, enhanced regional coordination and optimized policy support mechanisms should be prioritized to promote more balanced and sustainable urban vitality across China’s border regions.

### 4.2 Analysis of internal obstacle factors

This research measured the main internal factors hindering the vitality development of Chinese border cities in 2000, 2010, 2019, and 2022 based on the obstacle model, and selected the top 10 influencing factors (Table 4) for further analysis the key drivers affecting urban vitality. From the perspective of single-indicator obstacles, C4 (actual utilized foreign capital), C3 (international tourism receipts), C1 (import and export volume), C2 (import and export volume/GDP), and A2 (local general public budget revenue) show no significant changes. B1 (social fixed assets investment), D4 (number of college students enrolled in higher education institutions), D2 (technology expenditure), A4 (total profit of large-scale enterprises), and B3 (total retail sales of consumer goods) exhibited obvious fluctuations in the impact intensity in different years. Among all border areas, C4 (actually utilized foreign capital) consistently showed the greatest obstacle to urban vitality and ranked first.

**Table 4:** Main obstacle factors of urban vitality in border areas of China in 2000—2022 (Unit:%).

2000		2010		2019		2022	
Obstacle factor	Obstacle degree	Obstacle factor	Obstacle degree	Obstacle factor	Obstacle degree	Obstacle factor	Obstacle degree
**C4**	7.1394	C4	7.0622	C4	8.0807	C4	8.1147
**C3**	6.4954	C3	6.6339	C3	6.1338	C3	7.5341
**C1**	5.2540	C1	5.4609	C1	5.6183	C1	5.5076
**C2**	3.5623	C2	3.7563	C2	4.0174	C2	4.0183
**B1**	3.0805	D4	3.0371	D4	3.0970	D4	2.8832
**D4**	3.0210	B1	3.0085	A4	2.9057	B1	2.6841
**D2**	2.6987	A4	2.7383	B1	2.7634	A4	2.6614
**A4**	2.6851	B3	2.6090	D2	2.5162	B3	2.5299
**B3**	2.5850	D2	2.5496	B3	2.4778	D2	2.3767
**A2**	2.2784	A2	2.1759	A2	2.0357	A2	1.9006

**Fig 6:**
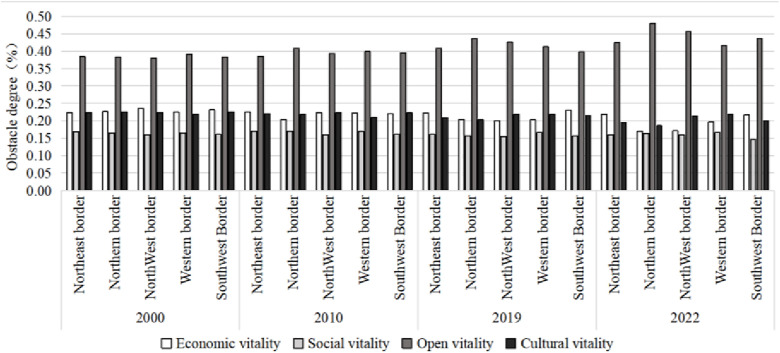
Obstacle degree of urban vitality system in border areas of China in 2000–2022.

From the perspective of the obstacle degree of the urban vitality system in border areas (Fig 6), from 2000 to 2022, the impact of border area opening-up on urban vitality has consistently been greater than the impact of social, cultural, and economic vitality, indicating that openess vitality is the primary constraint on the vitality of border cities. This is due to significant constraints in areas such as cross-border trade, regional cooperation, and policy, which have hindered the connectivity and collaboration of border regions with the outside world. The constraints of other vitality dimensions vary across different border regions: In the Northeast and Southwest border regions, the constraint on economic vitality has gradually increased, these regions have relatively weak economic foundations, low industrialization levels, and inadequate infrastructure, resulting in limited overall economic growth potential. In the Northern, Northwestern, and Western border regions, the constraint on cultural vitality has significantly increased, these regions feature distinct ethnic cultures, and the integration of traditional culture with modern culture has been obstructed, impeding cultural innovation and diversity, which, in turn, affects the development of their cultural vitality. The impact of social vitality on all border regions remains relatively stable, due to improvements in social infrastructure and government investment in education, healthcare, and public services, ensuring basic social vitality.

In summary, the constraints on different vitality dimensions vary across the border regions, openess vitality plays a crucial role in determining the vitality of border cities and should be a focal point of attention. Furthermore, efforts in policy optimization, infrastructure development, cultural exchange, and social progress should be made to enhance the overall vitality of border regions.

### 4.3 Analysis of external driving factors

Through a comprehensive literature review and based on the understanding of urban vitality, this study selects external influencing factors, including neighboring countries’ development strength [42], market opportunities [42], border policies [38], innovation capabilities [43], ecological environment [44] and urban size [21].

The development strength of neighboring countries was represented by a composite index, using import-export trade and neighboring country GDP as proxy variables. Market opportunities were represented by the ratio of neighboring countries’ GDP per Capita to regional GDP. Border policies were assigned values based on the level of ports (approved by the state council: class I ports for opening up to the outside world, key development and opening experimental zones, border economic cooperation zones, and border economic cooperation zones), with policy conditions assigned a score from 1 to 4, corresponding to 1 point for each level. Innovation capacity, ecological environment and urban scale were represented by per Capita water resources, patent authorizations, and population density, respectively.

Through collinearity diagnostics of the selected variables, the tolerances of all variables were greater than 0.1, and the VIF values were less than 10, indicating that there was no collinearity problem among the variables [46].

The results of multiple linear regression (Table 5) indicate that border policies and innovation capabilities had a significant impact on the vitality of border cities over different periods. In 2000, the most important factors affecting the vitality of cities in China’s border areas were innovation capabilities, border policies and ecological environment. The country proposed the strategy of prospering the frontier and enriching the people, along with the development of the western region, which provided development opportunities for enhancing the vitality of cities in border areas. The impact of the ecological environment on urban vitality was negatively correlated, indicating that natural conditions restricted the development of cities in border areas. The country should focus on the regions with weak ecological environment and strengthen their infrastructure construction. In 2010, the main factors affecting the vitality were innovation capability, neighboring country strength, border policies and market opportunities. Due to the financial crisis in 2008, the economic development of cities in border areas was unstable. Therefore, an open market could better promote the development of these cities. For example, the comprehensive launch of the China-ASEAN Free Trade Area effectively boosted the vitality of cities in China’s southwestern border areas. In 2019, the main factors affecting the vitality of cities in China’s border areas were border policies, innovation capabilities and urban size. The “Belt and Road” initiative, a strategic innovation of China’s development, provided a better platform and foundation for the development of cities in border areas, promoting these areas to become the forefront of opening up. At the same time, population growth and talent aggregation also effectively drove the improvement of urban vitality. In 2022, the main factors affecting the vitality of cities in China’s border areas were innovation capability, border policies, and market opportunities. Due to the global spread of COVID-19, economic development slowed, and the impact of market opportunities on the vitality of cities became negatively correlated, with significant constraints.

**Table 5:** Multiple linear regression results of urban vitality in border regions of China in 2000–2022.

Year	Influence factor	Proxy variables	B	T	P	VIF
**2000**	Innovation capabilities	Number of patent authorizations	0.538	4.186	0.000***	1.276
	Ecological environment	Per capita water resources	−0.262	−2.139	0.039*	1.162
	Border policies	Assignment	0.396	3.139	0.003**	1.229
**2010**	Innovation capabilities	Number of patent authorizations	0.749	7.598	0.000***	1.382
	Strength of neighboring countries	Composite index	−0.213	−2.204	0.034*	1.334
	Border policies	Assignment	0.204	2.170	0.036*	1.259
	Market opportunities	GDP per Capita of neighboring countries/GDP per Capita of regions	0.210	2.072	0.045*	1.334
**2019**	Border policies	Assignment	0.362	2.898	0.006**	1.158
	Innovation capabilities	Number of patent authorizations	0.283	2.255	0.03*	1.171
	Urban size	population density	0.309	2.273	0.029*	1.37
**2022**	Innovation capabilities	Number of patent authorizations	0.813	10.883	0.000***	1.2
	Market opportunities	GDP per Capita of neighboring countries/GDP per Capita of regions	−0.296	−2.087	0.044*	4.326
	Border policies	Assignment	0.238	3.151	0.003**	1.228

## 5. Discussion

### 5.1 Development suggestions for enhancing urban vitality in various border areas

From 2000 to 2022, the overall vitality of Chinese border areas improved, but the growth rate varied across different regions, with slight changes in the development stage of urban vitality.

In the northeastern border area, the average urban vitality was 0.04, 0.11, 0.12, and 0.11 in 2000, 2010, 2019, and 2022, respectively. The growth rate was relatively fast from 2000 to 2010; but after 2010, the growth rate declined significantly due to population loss, pressure for industrial transformation, a high proportion of resource-based industries, and a single economic structure. For the future development, this region should strengthen the construction of the China-Russia and China-North Korea border trade zones, enhance the functions of cross-border logistics hubs such as Hunchun and Suifenhe, promote the development of cooperative industrial parks with Russia, and form special industrial clusters such as energy processing, wood deep processing, and automotive parts manufacturing. Additionally, the northeastern border areas could promote the development of high-tech industries such as equipment manufacturing, artificial intelligence, and new materials, increase industrial added value, develop clean energy industries such as wind energy, solar energy and biomass energy, and reduce dependence on traditional heavy industry. Finally, leveraging ice and snow resources could provide opportunities for the development of winter tourism, outdoor sports, and wellness industries.

In the northern border area, urban vitality showed 0.03, 0.13, 0.19 and 0.18 in 2000, 2010, 2019, and 2022, respectively, The growth rate remained stable from 2010 to 2019, but declined in 2019 due to the impact of the epidemic. The region’s ecological environment is fragile due to its predominance of grasslands and deserts, and the reliance on a resource-based economy is high. Logistics and infrastructure construction are insufficient, and the population density is low. In the future, the region can rely on ports such as Erenhot, Manzhouli, and Ceke to accelerate the establishment of trade and economic cooperation zones. Furthermore, it could develop industries for desert management and grassland ecological restoration, such as ecotourism, green planting, and modernization of grassland animal husbandry, and guide the development of new energy industries like wind power and photovoltaic to form a sustainable development model. Supports such as tax incentives and housing subsidies could also attract young talents and entrepreneurs, which can promote the urban population growth.

The urban vitality in the northwest border area has increased year by year, and some cities have improved rapidly. The region borders many central Asian countries, and is the core area of the “Belt and Road”. It has sparse population but rich resources. By leveraging ports such as Khorgos and Kashgar, the region could accelerate the construction of cross-border e-commerce and logistics hubs, develop cross-border finance, commerce, cultural industries, and promote international cooperation. Based on its rich oil, natural gas, and mineral resources, the region could develop high-end refining, chemical new materials, intelligent manufacturing, and other industrial chains, as well as the deep processing of characteristic agricultural products like red dates, walnuts, and wine, enhancing their brand influence.

The improvement of urban vitality in the western border area has been relatively slow in the studied period, mainly due to the fragile ecological environment, weak economic foundation, and incomplete infrastructure construction limited by natural conditions. To enhance urban vitality, the region could encourage the development of special industries such as Tibetan medicine, yak dairy products, and organic agricultural products. Moreover, strengthening transportation infrastructures such as roads and airports, also could improve urban accessibility then boost the local development.

The urban vitality of the southwestern border region was 0.02, 0.08, 0.22 and 0.18 in 2000, 2010, 2019, and 2022, respectively. This region experienced the fastest improvement, especially from 2010 to 2019. This area has rich biodiversity and prominent tourism resources, and enjoys close economic ties with ASEAN countries. However, the local industries are primarily agriculture, tourism-based, and border trade, with a relatively weak industrial foundation.. In the future, the region could focus on constructing port economic zones such as Pingxiang, Dongxing and Ruili, developing cross-border e-commerce and trade logistics, and strengthening transportation infrastructure to enhance local development. Additionally, benefiting from the unique weather condition, high value-added agricultural industries such as tea, rubber, flowers, and tropical fruits could be also promoted.

Our results provide empirical evidence that urban vitality in China’s border regions has steadily increased over the past two decades, yet significant spatial disparities and internal constraints persist. Moreover, comparative cases also reveal diverse growth patterns and barriers. For example, Chongzuo’s transformation was driven by its port density, logistical advantages, and deep ASEAN ties, whereas Ali’s stagnation is rooted in geographic isolation and inadequate policy targeting. These cases suggest that a one-size-fits-all approach is insufficient. Localized development strategies-such as supporting clean energy clusters in the northwest border, or enhancing education and healthcare access in western border-are essential for narrowing the vitality gap.

### 5.2 Comparing international research on the vitality of border cities

Through a comparative analysis of China’s border cities and border cities in other parts of the world, we can identify some common key factors; however, the development of China’s border cities has its unique experiences and context. The specific analysis is as follows:

(1) Geo-Economics and Cross-Border Cooperation: A universal consensus in global research is that geo-economics and cross-border cooperation are crucial to the development of border cities. For instance, cooperation within the Schengen Area and the establishment of cross-border economic zones within the European Union have significantly enhanced the economic vitality of border cities in Europe [47]. Similarly, in North America, the North American Free Trade Agreement (NAFTA) has played a pivotal role in promoting economic linkages between neighboring border cities, fostering shared prosperity.
(2) Policy Intervention and Innovation: An important theme emerging from global studies is the irreplaceable role of policy intervention and innovation in promoting the vitality of border cities. China’s Belt and Road Initiative and border prosperity policies—such as the “prospering the border and enriching the people” initiative—serve as prime examples of how state-led policies shape the development of border regions. These are comparable to regional initiatives in Europe, such as the INTERREG plan, which fosters cross-border cooperation by emphasizing bottom-up regional development [48]. However, while policies like the EU’s cross-border regional cooperation plans support local and regional collaboration, China’s model relies more on top-down national strategies that direct urban growth and cross-border engagement. Similarly, the North American model often emphasizes multilateral trade agreements to stimulate economic cooperation across borders [49].
(3) Distinct Development Models in the European Union, North America, and China: The development model of border cities in the European Union and North America largely follows a bottom-up regional cooperation approach. In the EU, many border cities coordinate development by establishing joint management institutions and decision-making mechanisms to enhance cross-border cooperation [50]. Conversely, China’s border cities have tended to rely more on top-down national strategies, such as the implementation of the “Belt and Road”Initiative and policies to prosper the border regions. This reflects a centralized governance model where national policies are the driving force behind urban vitality, rather than the local collaborative efforts seen in other regions.

### 5.3 Research contributions

#### 5.3.1 Academic contributions.

By considering the concept of urban vitality and the unique characteristics of border areas, this study has proposed a definition for the urban vitality of border regions. Unlike traditional research on urban vitality, this study highlights that border cities have been constrained by their unique geographical locations, policy environment, and conditions for opening up to the outside world. As a result, their vitality has exhibited significant spatial polarization, resource dependence, and other characteristics. Therefore, the vitality of border areas has not only been reflected in conventional dimensions such as population mobility, economic prosperity and industrial diversity, but also needs to be measured and analyzed in conjunction with border-specific factors, such as cross-border trade and the level of port economic development.

Additionally, this study has developed a more targeted evaluation system for the vitality of border cities, taking into account their unique characteristics. The framework has been structured around four aspects: economic vitality, social vitality, openness vitality and cultural vitality, which provides a comprehensive reflection of the actual vitality of border areas. Based on this, a detailed assessment framework of urban vitality for border areas has been completed.

Finally, to examine the internal factors and external influences affecting the urban vitality of border cities, the obstacle degree model and multiple regression analysis has been applied. This has not only enriched the theoretical system of urban vitality research, but also provided scientific basis for policy formulation and development planning in border areas.

#### 5.3.2 Practical contributions.

Firstly, this rearch has provided the government with scientific assessment tools for the vitality of border cities, which has been helpful to policy making. The government has been able to quantify the vitality level of various border cities using this framework, identify cities with bottlenecks in resource utilization, industrial development, social services, etc., and formulate targeted management policies.

Secondly, this study has identified the key factors that affect the vitality of border cities in different regions and at different times, and has emphasized the importance of border policies, innovation capabilities, and other factors on urban vitality. The research results have assisted governments at all levels formulating targeted policies based on regional characteristics, optimizing resource allocation, and promoting high-quality economic and social development in border areas.

Furthermore, this study has provided valuable experience and reference for other countries and regions worldwide. With the promotion of the “Belt and Road” initiative, the importance of border cities in the process of globalization has become increasingly prominent, and the openness and development potential of border cities have become particularly important. By optimizing the infrastructure construction of border cities and promoting cross-border trade and economic cooperation, the government has been able to enhance the competitiveness of border cities in the global economy, provide stable support for the cooperation of countries along the “Belt and Road”, and offer important reference points for other countries’ border regions in formulating cross-border cooperation and regional development policies.

### 5.4 Limitations

This research aims to provide a new perspective for the study of urban vitality, but due to some limitations in research data, there are still some shortcomings. For instance, treaties between countries, borders, and cities often reflect the consensus of the political elite, while the consensus of local residents, economic activities and cross regional economic interactions may be influenced by more implicit and insufficiently quantified factors, especially in conflict prone areas. These factors are often not reflected in existing official data, and this limits a comprehensive understanding of economic interactions between regions. Due to the lack of such detailed information, the findings of this study may have certain limitations across different countries or regions. Therefore, future research should expand the data coverage, particularly in situations involving conflict zones or special economic relationships, to include a broader range of economic data and socio-economic interaction factors, providing a more comprehensive and objective presentation of the diversity and complexity of global economic relationships.

While the ecological environment was found to have a negative impact on vitality in 2000, its key role in urban vitality construction has not been fully explored. Border cities located in ecologically sensitive areas-such as deserts (Inner Mongolia) or highlands (Tibet)-face infrastructure and population density limitations. However, these regions also possess untapped potential for green economy initiatives, including renewable energy, eco-tourism, and conservation-linked livelihoods. Future research could integrate environmental indicators such as carbon emissions, green infrastructure coverage, or biodiversity metrics to better assess “sustainable vitality”.

Moreover, urban vitality in border areas is shaped by a complex interplay of economic, geopolitical, cultural, and ecological factors. This necessitates interdisciplinary approaches combining urban geography, development economics, political science, and planning. In the future, spatial econometric models, situational simulations, or qualitative case studies can be introduced to deepen mechanism analysis and improve the theoretical system of “border city vitality”.

## 6. Conclusion

This research focused on 45 prefecture-level cities in China’s border areas, constructed an urban vitality index system consisting of four dimensions: economic vitality, social vitality, openness vitality and cultural vitality, and examined the spatiotemporal differentiation and influencing factors of urban vitality in urban border areas, The main conclusions were as follows:

(1) In terms of temporal evolution, the level of urban vitality in China’s border areas has been steadily increasing. Cities with high vitality were mainly concentrated in resource-based cities with high port levels and strong economic foundations.
(2) In terms of spatial features, high-vitality areas were relatively dispersed, forming a spatial polarization pattern centered around Mudanjiang City in the northeast border area, Baotou City in the northern border area, Bortala Mongolian Autonomous Prefecture in the northwest border area, and Chongzuo City in the southwest border area.
(3) In terms of influencing factors, from the perspective of internal obstacles, the overall vitality of border cities was more significantly influenced by openness vitality than by cultural vitality, social vitality, or economic vitality. Between 2000–2022, the northern border region experienced the largest impact from openness vitality, followed by the northwest, southwest, northeast, and western border regions. From an external perspective, border policies and innovation capabilities have consistently been the strongest external factors affecting the vitality of China’s border cities. The development strength, market opportunities, ecological environment, and urban size of neighboring countries exerted varying degrees of influence over time.

In summary, cities benefiting from national-level policy support-such as border economic cooperation zones and the RCEP-have demonstrated greater vitality. In the future, attention should be given to the dynamic nature of cross-border cooperation and its impact on the vitality of border cities. Further simulations should be conducted on how trade agreements, infrastructure development, and geopolitical changes affect urban openness and overall vitality, in order to provide deeper theoretical support and policy guidance for the sustainable development of border cities.

## Supporting information

S1 Data Evaluation indicators and multiple linear regression data. (XLS)

## References

[1] Lai Y, Huang Q. The rise of border cities in the context of globalization. J Borderland Stud. 2020;35:159–75.

[2] Sun J, Cun Y. The conditions, values, and policy recommendations for the construction of China’s border urban system. Gansu Soc Sci. 2022;4:194–203.

[3] Tong M. How urban fabric can help sustain the vitality of cities. Urban Plan Forum. 2014;3:85–96.

[4] Lang W, Webster JC. Urban vitality in compact cities: seeing Hong Kong through Kelvin Lynch’s lens. Urban Planning Inter. 2017;32:28–33.

[5] Rosière S, Jones R. Border regions in global economic integration. Geopolitics. 2020;25:812–33.

[6] Sohn C, Giffinge R, Miletić A. Cross-border city regions and regional development: insights from European case studies. Reg Stud. 2021;55:681–94.

[7] Ehlers N. Borders in globalization: a research agenda for border studies in times of crisis. J Borderlands Stud. 2020;35:497–511.

[8] Maas PR. Towards a theory of urban vitality. Ottawa: University of British Columbia; 1984.

[9] Lynch K. Good city form. Cambridge: MIT Press; 1984.

[10] Bentley I. Responsive environments. London: Routledge; 1985.

[11] Huang B, Zhou YL, Li ZG. Evaluating and characterizing urban vibrancy using spatial big data: Shanghai as a case study. Environment and Planning B: Urban Analytics and City Science. 2020;47:1543–59.

[12] Wang JG. Inclusiveness and sharing, explicit and implicit mutual learning, livability predicted: historical prospect and contemporary creation of urban vitality. City Plan Rev. 2019;43:9–16.

[13] Katz P. The new urbanism: toward an architecture of community. New York: Mc Graw-Hill; 1993.

[14] Jin YJ. Study on urban economic vitality index in China. Scientia Geographica Sinica. 2007;27(1):9–16.

[15] Yadav R, Seth A, Dembla N. Optimizing crop yield prediction: data-driven analysis and machine learning modeling using USDA datasets. Curr Agri Res J. 2024;12(1):272–85.

[16] Shu TH, Ren YT, Shen LY. Study on spatial heterogeneity of consumption vibrancy and its driving factors in large city: a case of Chengdu city. Urban Develop Stud. 2020;27:16–20.

[17] Wang N, Wu JS, Li S. Spatial features of urban vitality and the impact of built environment on them based on multi-source data: a case study of Shenzhen. Trop Geo. 2021;41:1280–90.

[18] China Academy of Urban Planning and Design. Assessment report on prosperity and vitality of Chinese cities in 2022. Beijing, China: China Academy of Urban Planning and Design; 2022.

[19] Lan F, Gong XY, Da HL. How do population inflow and social infrastructure affect urban vitality? Evidence from 35 large-and medium-sized cities in China. Cities. 2020;100:102454.

[20] Li ZY, Guo YY, Han ZZ. Comparative research on statistical measurement of city vitality. World Surv Res. 2021;(8):74–80.

[21] Ta N, Zeng YT, Zhu QY. Relationship between built environment and urban vitality in Shanghai downtown area based on big data. Scientia Geographica Sinica. 2020;40:60–8.

[22] Si R, Lin YY, Xiao ZP. Spatio-temporal analysis of built environment and street vitality relationship based on street-level imagery: a case study of Futian District, Shenzhen. Scientia Geographica Sinica. 2021;41:1536–45.

[23] Gehl J. Life between buildings: using public space. Washington, USA: Island Press; 1987.

[24] Jacobs J. The death and life of great American cities. New York: Random House; 1961.

[25] Jiang DF. The theory of city form vitality. Nanjing: Southeast University Press; 2007.

[26] Beltran Rodriguez M, Simon M. Conceptualizing conviviality in urban landscapes. Athens J Archit. 2015;1:311–26.

[27] Pentland A. Social physics: how good ideas spread - the lessons from a new science. Penguin Press; 2014.

[28] Sohn C. Modelling cross-border integration: the role of borders as a resource. Geopolitics. 2014;19(3):587–608.

[29] Durand F, Decoville A. Exploring cross-border integration in Europe: how do populations cross borders and perceive border barriers?. Euro Plan Stud. 2018;26(1):23–45.

[30] Newman D. The lines that continue to separate us: borders in our `borderless’ world. Prog Hum Geogr. 2006;30(2):143–61.

[31] Brunet-Jailly E. Theorizing borders: an interdisciplinary perspective. Geopolitics. 2005;10(4):633–49.

[32] Chen X, Liao S, Han X. Border cities and regional integration in China: an overview. Asia Pacific Viewpoint. 2020;61(2):237–51.

[33] Shen J, Ma L. China’s regional development and spatial restructuring. Routledge; 2021.

[34] Lopes MN, Camanho AS. Public green space use and consequences on urban vitality: an assessment of European cities. Social Indicators Res. 2013;113:751–67.

[35] Wang YZ. Small and medium-sized enterprises and economic vitality. China Indust Econ. 1995;(10):30–6.

[36] Cao Y, Ge YJ, Ma T. The spatiotemporal evolution and evaluation of comprehensive national power of China and surrounding countries since 21st century. Econ Geo. 2018;(38):45–54.

[37] Dong H. Ethical reflection on the generation of social vitality practice. J Hubei Univ (Philosophy and Social Science). 2010;37:33–8.

[38] Cheng Y, Liu H, Gong PP. Spatial disparity and its major influencing factors of export-oriented economy in Chinese border area. Econ Geo. 2016;36:19–26.

[39] Chen MH, Wang Z, Xie LX. The spatiotemporal pattern evolution and formation mechanism of high-quality development in Central China. Acta Geographica Sinica. 2023;78:859–76.

[40] Tian L. The analysis of spatial-temporal pattern and influencing factors of border tourism. Ecol Econ. 2023;39(07):149–56.

[41] Ren T, Liu JS. Spatial dimension and obstacle analysis of tourism competitiveness in Jilin Province. Econ Geo. 2011;31:2138–43.

[42] Song T, Cheng Y, Liu WD. The spatial disparity and impact mechanism of geo-economy in the border areas of China. Acta Geographica Sinica. 2017;72:1731–45.

[43] Yuan CP, Du Y. A study of the impact of the digital economy on urban resilience: based on empirical evidence from 277 cities during the epidemic. Urban Develop Stud. 2021;30:51–7.

[44] Zhao HB, Yue L, Liu YX. Spatial-temporal pattern and obstacle factors of urban residents’ quality of life in the Yellow River Basin under the background of high quality development. Scientia Geographica Sinica. 2021;41:1303–13.

[45] Leng JF, Gao X, Zhu JP. Application of multiple linear regression and statistical forecasting model. Stat Decision. 2016;7:82–5.

[46] Chen T, Bao AM, Guo H. Ecological vulnerability assessment for a transboundary basin in Central Asia and its spatiotemporal characteristics analysis: taking AmuDarya river basin as an example. J Nat Res. 2019;34:2643–57.

[47] Sohn C. Navigating borders’ multiplicity: the critical potential of border studies. Polit Geo. 2020;82:102238.

[48] Perkmann M. Cross-border regions in europe: significance and drivers of regional cross-border co-operation. Euro Urban Reg Stud. 2003;10:153–71.

[49] Hansen N. Border cities and urban development: the case of the US-Mexico border. J Borderlands Stud. 2021;36:22–39.

[50] Johnson C, DeVoretz D. The economics of EU border regions. J Econ Geo. 2021;15:655–79.

